# Supplementary material for: Unconventional supercurrent phase in Ising superconductor Josephson junction with atomically thin magnetic insulator
Source: Nat Commun. 2021 Sep 9;12:5332. doi: 10.1038/s41467-021-25608-1 (PMC8429564; doi:10.1038/s41467-021-25608-1)
Supplement: Supplementary file 1 — Supplementary Information [file 41467_2021_25608_MOESM1_ESM.pdf]

Supplementary information

for

Unconventional supercurrent phase in Ising superconductor Josephson junction with atomically thin magnetic insulator

H. Idzuchi[1], F. Pientka[1,2], K.-F. Huang[1], K. Harada[3], Ö. Gül[1], Y. J. Shin[1], L. T. Nguyen[4], N. H. Jo[5,6], D. Shindo[3], R. J. Cava[4], P. C. Canfield[5,6], and P. Kim[1*]

[1] *Department of Physics, Harvard University, Cambridge, MA 02138, USA*

[2] *Institut für Theoretische Physik, Goethe-Universität, 60438 Frankfurt am Main, Germany*

[3] *Center for Emergent Matter Science (CEMS),RIKEN, Wako, Saitama 351-0198, Japan*

[4] *Department of Chemistry, Princeton University, Princeton, NJ 08540, USA*

[5] *Department of Physics and Astronomy, Iowa State University, Ames, IA 50011, USA*

[6] *Ames Laboratory, Iowa State University, Ames, IA 50011, USA*

Supplementary Note 1. Magnetic-field hysteresis of critical current in the junction with thick $Cr_2Ge_2Te_6$ barrier.

We have measured the critical current of $NbSe_2/Cr_2Ge_2Te_6/NbSe_2$ junction with a 6-ML $Cr_2Ge_2Te_6$ barrier as a function of magnetic field perpendicular to the 2D layer. The data were taken both for decreasing (the red lines and arrows) and increasing (the black lines and arrows) magnetic fields, as shown in Figs. 2a-2b. The magnetic-field hysteresis of critical current is clearly present. The characteristic field strength of the hysteresis in critical current differs from the hysteresis of magnetization in $Cr_2Ge_2Te_6$ alone, as shown in the magnetization curves measured by SQUID in Fig. 2c. To study the origin of the hysteresis, we have measured the magnetic structure of $Cr_2Ge_2Te_6$ by means of a transmission electron microscope with Lorentz mode (Lorentz transmission electron microscopy, see Methods for details). We have observed stripe magnetic structures (Fig. 2d) and bubble-like ones (Fig. 2e) in the Lorentz micrographs. We did not see the pattern when the sample is placed perpendicular to the electron beam; the pattern appears when the sample is tilted, indicating that the magnetic structure exhibits out-of-plane magnetic moment. The stripe pattern appears dominantly, and the bubble-like one appeared in the same sample after a different thermal cycle, implying the latter one is in a metastable state.

Supplementary Fig. 1a shows the possible magnetic configuration in $NbSe_2/Cr_2Ge_2Te_6(6ML)/NbSe_2$

junction under perpendicular magnetic field. When the field is large, dense vortices in $NbSe_2$ can form a uniform magnetic structure in $Cr_2Ge_2Te_6$. Decreasing the strength of the field decreases the density of vortices. The vortex array increases the energy cost to form the stripe pattern, and the magnetic structure with the hexagonal-like pattern becomes more stable (Supplementary Fig. 1c). At small external magnetic fields, the magnetic field is shielded by $NbSe_2$ (Meissner effect), and the magnetic domain of $Cr_2Ge_2Te_6$ should prefer the stripe pattern (Supplementary Fig.1d). At increased fields, vortices are formed in the $NbSe_2$ layer. The energy cost to form bubble-like pattern decreases while there is an energy barrier to switch between the two types of the structures. Finally, increasing the field further leads to the uniform magnetization state again (Supplementary Fig. 1b).

As shown in Supplementary Fig. 1, one possible scenario to explain our experimental observation (Fig. 2) is to consider the interaction between the magnetic domain in $Cr_2Ge_2Te_6$ and vortex lattice in $NbSe_2$. Particularly, forming a stripe magnetic domain pattern in the $Cr_2Ge_2Te_6$ layer near the domain reversal can cost extra energy in vortex configuration in the superconductor. Thus, during the transition from the triangular bubble domain to the more stable stripe domain, one expects a reduction of Josephson free energy, resulting in the step in $I_C(H)$.

The magnetization of bulk $Cr_2Ge_2Te_6$ does not show notable hysteresis (Fig. 2c), which is consistent with earlier reports [1,2]. This indicates a small magnetic structure, which is averaged out in the magnetization measurements, may play a role in switching current of Josephson junction. Further, in $NbSe_2$, the lower critical field B_{C1} is found to be 19 mT and 10 mT for the out-of-plane and in-plane fields, respectively [3]. The low field behavior of Figs. 2a and 2b are attributed to this difference.

We also measured the field dependence of the voltage at a high bias (much larger than the critical current). This measurement did not show notable hysteresis, as shown in Supplementary Fig. 2.

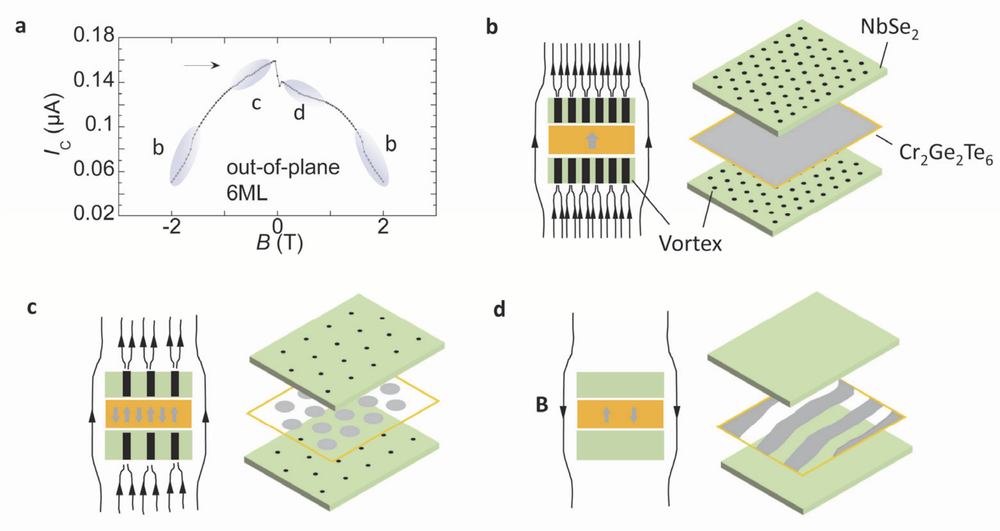

Supplementary Fig.1| Possible configuration of magnetic domain structure in $Cr_2Ge_2Te_6$ with vortices in $NbSe_2$. a, The critical current of $NbSe_2/Cr_2Ge_2Te_6/NbSe_2$ junction with a 6-ML $Cr_2Ge_2Te_6$ barrier at the temperature of 0.3 K as a function of magnetic field perpendicular to the 2D layer. Arrows indicate the sweep direction. The shaded areas labeled with b, c and d indicate the field regions corresponding to the configurations of (**b**) – (**d**). **b-d,** Preferential magnetic structure in $Cr_2Ge_2Te_6$ with vortex pattern in $NbSe_2$. Green and orange rectangles represent $NbSe_2$ and $Cr_2Ge_2Te_6$, respectively. Black rectangles and dots indicate superconducting vortices. The black lines with triangles (arrows) indicate magnetic field lines. The gray arrows in the orange box indicate magnetization direction of $Cr_2Ge_2Te_6$. Gray filled area in right panels indicate magnetic domain for up-magnetization in $Cr_2Ge_2Te_6$ (completely filled in (**b**), filled circles in (**c**), and filled stripes in (**d**)). As the vortex can penetrate and focus the magnetic field near its core, the part of the $Cr_2Ge_2Te_6$ exposed to the strong fields should be magnetized along that direction. (**d**) depicts a Meissner phase.

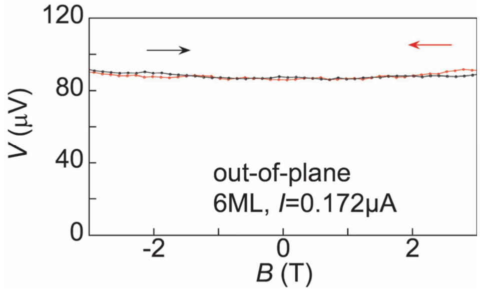

Supplementary Fig.2| The field-dependence of the voltage at high bias for the device $NbSe_2/Cr_2Ge_2Te_6/NbSe_2$. The device is the same as the one shown in Fig. 2a. The thickness of the $Cr_2Ge_2Te_6$ is 6ML. The temperature is at 0.3 K. The bias current is 0.172 μA and the field direction is out-of-plane.

Supplementary Note 2. Further characteristics of the SQUID with a 1ML $Cr_2Ge_2Te_6$ barrier junction.

In this section, we provide further characteristics of ϕ-junction. We studied the device of Fig. 3b after removing a part of the link in the arm in the SQUID connection. This allows us to see two-critical currents without SQUID oscillations. We have confirmed the device shows two different critical currents with one bias polarity, one for each sweep sequence (inset of Supplementary Fig. 3a). Four branches for switching current were characterized by varying current sweep sequences for both positive and negative bias directions (Supplementary Fig. 3a). Supplementary Fig. 3b shows the critical current under the in-plane field.

The probability of switching current was also characterized. Supplementary Fig. 4 shows the temperature variation at the field of -4.3 mT, which shows two switching currents (Supplementary Fig. 3b), indicating suppression of stochastic behavior with raising the temperature.

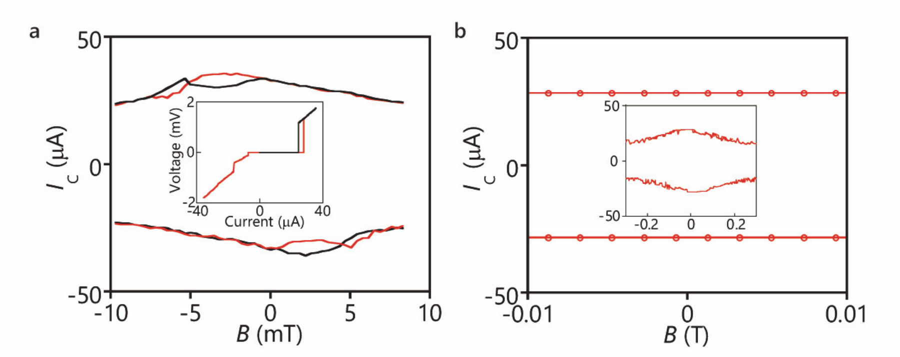

Supplementary Fig.3| Details of ϕ junction behavior. a, Four switching-current branches of ϕ-junction for the device shown in Fig. 3b, under out-of-plane magnetic field. The temperature is at 1.8 K. Inset shows the switching current when sweeping the current from negative values to positive (shown in red) is different from that when sweeping from positive values to zero (not shown) and then from zero to positive values (black). The magnetic field is at -2.2 mT and the temperature is at 2.1 K. **b**, switching currents under the in-plane magnetic field at the temperature of 1.4 K. Inset shows the ones for larger field range. Note that the background tilt in *I*-*V* curve is removed.

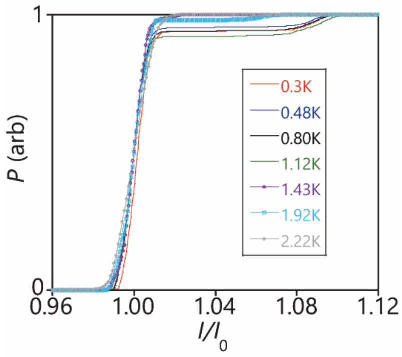

Supplementary Fig.4| The Switching probability *P* of ϕ-JJ with different temperatures at the out-of-plane magnetic field of -4.3 mT, where I_0 denotes the bias current at P = 0.5. The bias sweep is given from large positive through zero to large positive.

Supplementary Note 3. Fraunhofer pattern.

We have also studied the response of the in-plane magnetic field for devices besides the 6-ML one shown in Fig. 2. Supplementary Fig. 5 summarizes the response for $NbSe_2/Cr_2Ge_2Te_6/NbSe_2$ Josephson junction (JJ) as well as $NbSe_2/NbSe_2$ JJ made between two cleaved surfaces. We note the device without $Cr_2Ge_2Te_6$ was fabricated under ambient conditions, and the thickness of the top and bottom flakes are 12.7 nm and 100 nm, respectively. In addition to the clear Fraunhofer pattern in the $NbSe_2/NbSe_2$ junction, the devices with $Cr_2Ge_2Te_6$ show multiple peaks with the main peak around zero field. The second peaks are located at 0.14 T, 0.59 T and 0.79 T (averaged for positive and negative fields) for $NbSe_2/NbSe_2$ JJ, $NbSe_2/Cr_2Ge_2Te_6$(1ML)/$NbSe_2$ JJ, and $NbSe_2/Cr_2Ge_2Te_6$(6ML)/$NbSe_2$ JJ, respectively. The critical current in a Fraunhofer pattern is given by

$$I_C(X) = I_C(0)\left|\frac{sin(\pi X)}{\pi X}\right|,$$

with $X=\Phi/\Phi_0$, $\Phi=(d+2\lambda)LB$, where λ is the penetration length, d the thickness of the barrier layer, L the length of the junction and Φ_0 = 20.7 Gauss.μm^2. Inserting these values in the equation above provides with $d+2\lambda$ values of 6.8 nm, 6.2 nm, and 13.2 nm, which is reasonable values considering the nonideal device geometry and unavoidable inhomogeneity. Further irregularity can be caused by a field-induced development of magnetic texture, including sudden change due to domain wall nucleation.

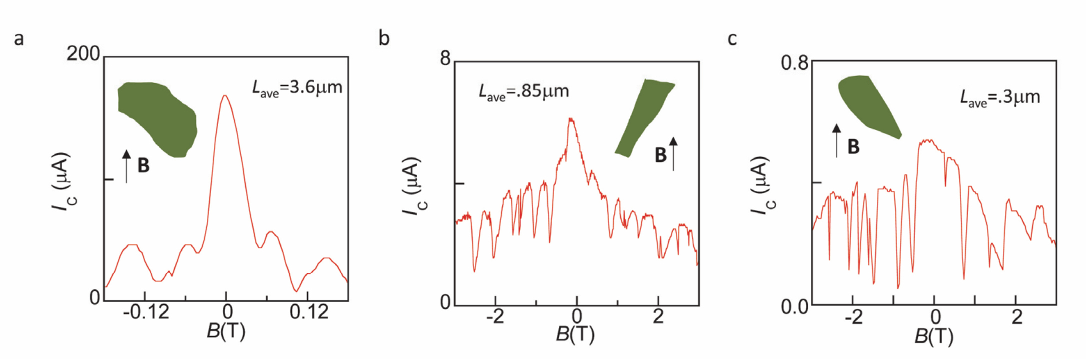

Supplementary Fig. 5| Fraunhofer pattern. In-plane field response of **a**, $NbSe_2/NbSe_2$ JJ. **b**, $NbSe_2/Cr_2Ge_2Te_6$(1ML)/$NbSe_2$ JJ. **c**, $NbSe_2/Cr_2Ge_2Te_6$(6ML)/$NbSe_2$ JJ. The temperature is at 1.6 K, 1.5 K, and 0.3 K from (**a**) to (**c**). The inset (green filled area) shows the shape of the junction with **B** field direction. L_{ave} indicates the average length of the junction along **B** field direction.

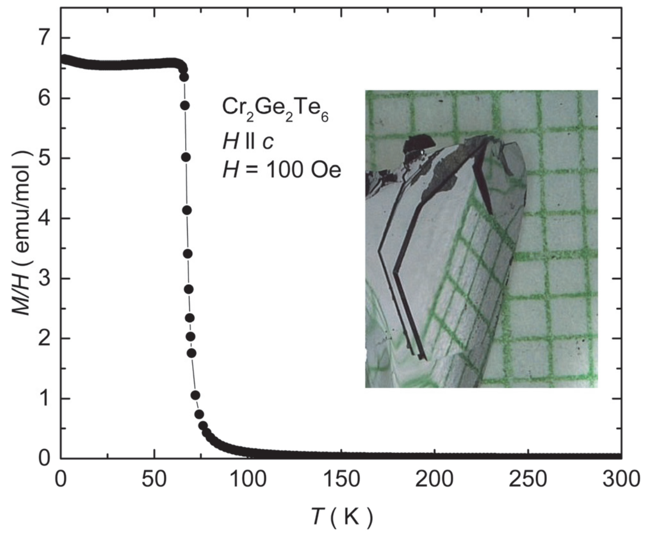

Supplementary Fig.6| $Cr_2Ge_2Te_6$ single crystal. Temperature variation of low field magnetization. Inset shows photograph of a faceted single crystal on mm-grid paper. The mirrored face of the crystal is reflecting the mm-grid in the image

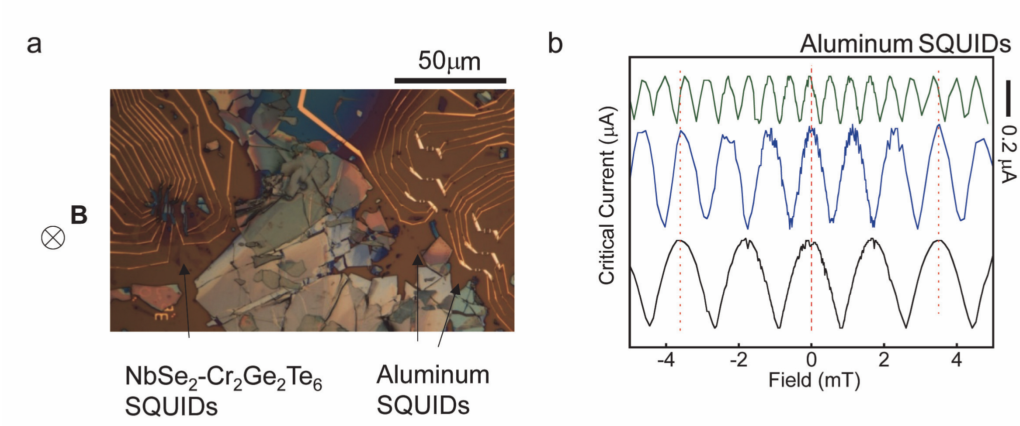

Supplementary Fig.7| Aluminum SQUIDs for magnetic field calibration. a, Optical micrograph of the $Cr_2Ge_2Te_6$-SQUID with aluminum SQUIDs for calibration of magnetic field. **b,** Critical-current oscillation for the three aluminum SQUIDs with different sensor areas, measured at $T = 0.3$ K. The plots are offset in the vertical direction for clarity.

Supplementary Note 4. Model Calculation

In order to corroborate our theoretical arguments in the main paper, we now evaluate the energy-phase relation of a magnetic-insulator Josephson junction based on a concrete microscopic model. We assume translational invariance in the plane perpendicular to the current, which flows along the *z* direction. Hence our model becomes effectively one-dimensional and the two in-plane momentum components $\mathbf{p}_{||}$ enter as parameters of the Hamiltonian. $NbSe_2$ has an inversion center between two layers that form the unit cell. As a consequence, each layer has strong spin-orbit coupling, which has opposite sign in even and odd layers. This leads to a large spin-orbit splitting Δ_{SOC} of the Fermi pockets around the the *K* and *K*' points within each layer, i.e., a locking of spin and layer degrees of freedom with spins quantized along the *z* direction [4]. Because of the weak interlayer hopping in $NbSe_2$, we can consider the electrons from even and odd layers as forming approximately independent bands. The Josephson effect is dominated by contributions from the first layer on each side of the magnetic barrier so we can focus only on the odd-layer band around the *K* and *K*' points. Any coupling from the other spin band at the *K* and *K*' points as well as from the pocket around the Γ point, which does not exhibit spin-layer locking, will yield an additional contribution to Josephson energy with energy minimum at ϕ=0, but will not change our general conclusions.

The strong spin-orbit coupling in the odd layers separates the two spins in momentum space around the *K* and *K*' points. Because the spin splitting exceeds the out-of-plane hopping strength, the bandwidth along the out-of-plane momentum p_z is smaller than the energy separation between different spin bands and we therefore expect the odd-layer Fermi surface sheets for spin up and down to occur at different in-plane momenta. This assumption is corroborated by DFT calculations [5]. Moreover, the spin splitting also exceeds pairing strength, $\Delta_{SOC} >> \Delta$, which means the Cooper pairs have spins quantized along the *z* axis. As a consequence, we can consider the Hamiltonian $H(\mathbf{p}_{||})$ at a momentum $\mathbf{p}_{||}$ on the Fermi surface as effectively spinless and ignore scattering to the spin which does not cross the Fermi level at that particular in-plane momentum.

i) Equal spins on both sides of the junction.

We assume for now an orientation of the $NbSe_2$ such that the Fermi surfaces with the same spin polarization on both sides of the junction are aligned in momentum space. We will later comment on the opposite case. The four Fermi surface sheets can labeled by ρ=±1 which equals +1 on the inner (outer) Fermi surface of the *K* (*K*') valley and -1 otherwise. The corresponding Hamiltonian in the continuum approximation is given by

$$H(p_{||}) = \left(\frac{p_z^2}{2m_z} + \frac{p_{||}^2}{2m_{||}} - \mu - \rho\Delta_{SO}\sigma_z\right)\tau_z + \delta(z)(V\tau_z + J\vec{\sigma}\cdot\mathbf{n}) + \Delta\cos[\phi(z)]\tau_x - \Delta\sin[\phi(z)]\tau_y, \quad \text{(S1)}$$

where μ is the chemical potential and the superconducting phase $\phi(z)$=sign(z)ϕ/2 jumps at z=0, the location of the barrier. The narrow magnetic barrier is modeled as a δ-function and includes potential scattering with strength *V* and magnetic scattering with strength *J*, which have both dimensions of velocity. The Hamiltonian has translational invariance in the plane and $p_{||}^2 = (p_x^2 + p_y^2)^{1/2}$ is therefore conserved. The spin-orbit splitting is $\Delta_{SO} > \ |p_{||}^2/2m_{||} -$

$\mu|$ such that system behaves as a superconductor for one spin and as an insulator for the other spin.

The scattering matrix of the barrier for states at the Fermi energy is given by

$$S_B = \begin{pmatrix} r & t \\ t & r \end{pmatrix} = \frac{1}{1-i\beta}\begin{pmatrix} i\beta & 1 \\ 1 & i\beta \end{pmatrix}, \tag{S2}$$

$$\beta = -\frac{(V\tau_z + J\vec{\sigma}\cdot\mathbf{n})}{v_z}, \tag{S3}$$

where v_z is the Fermi velocity in the z direction. At subgap energies, the superconducting leads have zero transmission and the corresponding scattering matrix is given by

$$S_{\mathrm{SC}} = \begin{pmatrix} r_{\mathrm{L}} & 0 \\ 0 & r_{\mathrm{R}} \end{pmatrix}, \tag{S4}$$

where $r_{\mathrm{L/R}}$ are the reflection matrix elements of the left and right lead, which depend on the spin of the incoming electrons. The reflection amplitudes can be approximated by

$$r_{\mathrm{L/R}} = \frac{(1+\rho\sigma_z)}{2}\tau_x e^{i\alpha \pm i\alpha\phi/2\tau_z} - e^{i\gamma}\frac{(1-\rho\sigma_z)}{2}, \tag{S5}$$

where the first term accounts for Andreev reflection of spin $\sigma_z = \rho$ with $\cos\alpha = E/\Delta$ and the second term describes normal reflection of spin $\sigma_z = -\rho$ with γ the reflection phase. We can obtain the energy spectrum of Andreev bound states in the junction from the condition

$$\det(1 - S_{\mathrm{SC}}S_{\mathrm{B}}) = 0. \tag{S6}$$

We now distinguish two cases depending on the magnetization direction in the magnetic insulator.

(i) *Out-of-plane magnetization.* For $\mathbf{n}=\hat{z}$ the Hamiltonian conserves spin and from Eq.(S6) we obtain in the $\sigma_z = \rho$ channel

$$\frac{V^2-J^2}{v_F^2} + \cos\phi = \left(1 + \frac{V^2-J^2}{v_F^2}\right)\cos(2\alpha) + \rho\frac{2J}{v_F}\sin(2\alpha), \tag{S7}$$

which yields four energy levels when both valleys are taken into account ($\rho=\pm1$). Closed analytical expressions for the energies can be easily obtained but are not very illuminating. We instead plot the result in Supplementary Fig. 8, which shows the energy spectrum as well as the ground state energy $E_{gs}^{\perp}(\phi)$ obtained by summing over the two negative energy solutions. For sufficiently large values of J, the Josephson junction has its ground state at $\phi = \pi$. For $V = 0$ the threshold for a π-junction is $J/v_{\mathrm{F}} \gtrsim 0.8$.

(ii) *In-plane magnetization.* For $\mathbf{n} = \hat{x}$ the spins can flip at the barrier. However, in the limit of large Δ_{SO}, the superconductors are essentially hard walls for electrons with spin $\sigma_z = -\rho$ and thus spin flip scattering is suppressed. Retaining only spin conserving scattering we obtain

$$\frac{V^2}{v_F^2} + \cos\phi = \left(1 + \frac{V^2}{v_F^2}\right)\cos(2\alpha), \tag{S8}$$

which is identical to Eq. (S7) with $J = 0$. One can explicitly verify this result by evaluating the full expression in Eq. (S6) and subsequently taking the limit $\gamma \rightarrow 0$, which corresponds to hard-wall reflection of spins $\sigma_z = -\rho$ in the limit $\Delta_{SO} \rightarrow \infty$. The valley degenerate energies are given by

$$E^{||}(\phi) = \pm\Delta\sqrt{\frac{1+2(V/v_F)^2+cos\phi}{2+2(V/v_F)^2}}, \tag{S9}$$

and the ground state energy $E^{||}_{gs}(\phi) = -2|E^{||}(\phi)|$ has a global minimum at $\phi = 0$.

(iii) *Magnetization with arbitrary angle.* In the case of a magnetization with both in-plane and out-of-plane components, $\mathbf{n} = (n_x, 0, n_z)$, the in-plane part, which leads to spin flip scattering, can again be ignored. This case therefore reduces to Eq.(S7) with the replacement $J \rightarrow Jn_z$, i.e., it smoothly interpolates between the cases (i) and (ii). As the magnetization is tilted towards the x - y plane, n_z becomes smaller effectively reducing the magnetic scattering amplitude. For sufficiently small out-of-plane magnetization the Josephson energy will always have a global minimum at ϕ=0.

The total Josephson energy depends on the spatial distribution of magnetic domains. If we assume that a fraction λ of the plane has an out-of-plane magnetization and the rest has an in-plane magnetization, the total ground state energy is

$$E_{gs}(\phi)=\lambda E^{\perp}_{gs}(\phi)+(1-\lambda)E^{||}_{gs}(\phi). \tag{S10}$$

Here we assume for simplicity a magnetization that is either in plane or out of plane, although a more complicated magnetic texture can be easily included. The ground state phase different for $\lambda = 0.9$ is plotted in Supplementary Fig. 9. There is clearly an extended region of parameter phase with a nonzero phase difference.

ii) Opposite spins on both sides of the junction.

In our model, we have so far assumed that the $NbSe_2$ Fermi surfaces have the same spin in the first layer on both sides of the junction. In the case when the spins are opposite, we can consider the same model with the spin orbit coupling replaced by $\Delta_{SOC} \rightarrow \Delta_{SOC}$ sgn(z). Cooper pair tunneling across the barrier now requires a spin flip, i.e., the magnetization must be in-plane. A similar calculation as above, shows that the ground state energy has a minimum at $\phi=\pi$ in a sizable fraction of parameter space. Note that in this case we need to choose $\gamma \neq 0$, because in the presence of a hard wall (i.e., γ=0) the wavefunction would have a node at the δ-function, which would render spin flips impossible and result in zero Josephson current.

When the magnetization is out of plane, our simple model yields no Josephson current because of spin conservation. In that case, other contributions, e.g., from the Γ point or due to spin mixing around the K and K' point,

would presumably lead to a ground state at zero phase. In conclusion, there is a similar competition between zero- and π-junctions that can conceivably result in an overall ground state at a nontrivial phase $\phi \neq 0, \pi$.

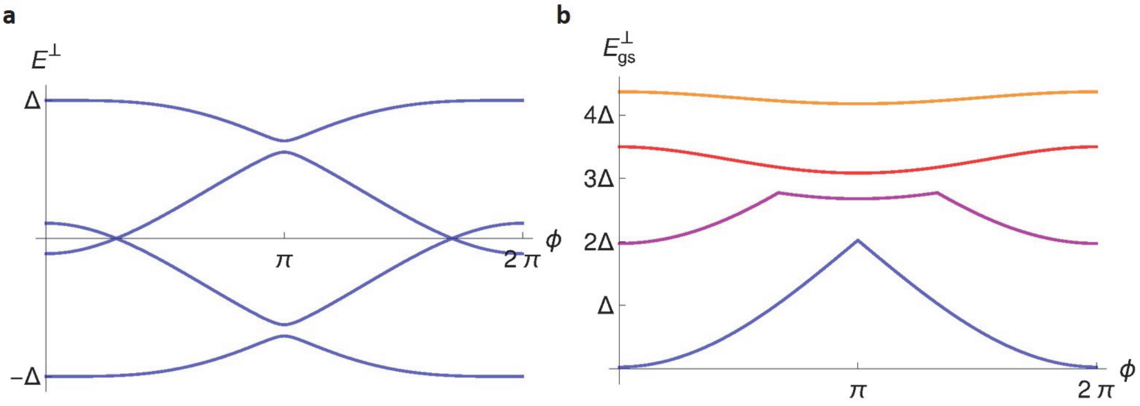

Supplementary Fig.8| a, Energy spectrum of Andreev bound states for out-of-plane magnetization with J/v_F=0.9 and V/v_F=0.1. **b**, Ground state energy for V=0 and J/v_F=0,0.5,1,2 from bottom to top (curves are shifted vertically for clarity).

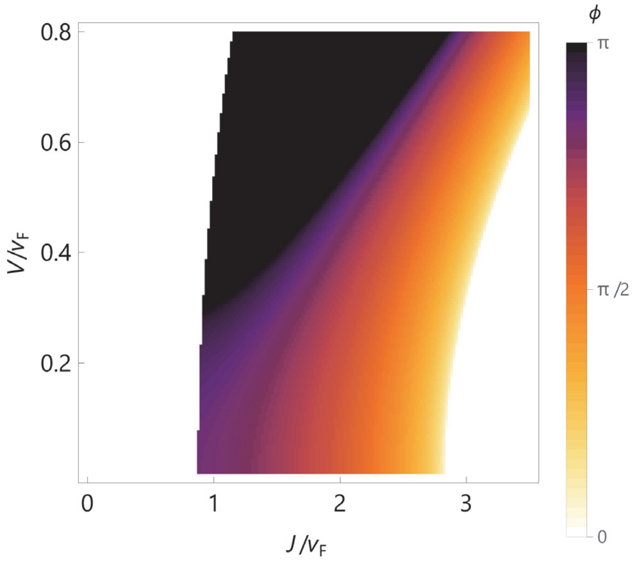

Supplementary Fig.9| Phase difference ϕ of the ground state energy in Eq.(S10) for λ=0.9. The white and black regions correspond to phases zero and π. The colored areas indicate a nontrivial phase difference across the junction in the ground state.

Supplementary References

1. Gong, C. *et al*. Discovery of intrinsic ferromagnetism in two-dimensional van der Waals crystals. *Nature* **546**, 265-269 (2017).
2. Carteaux, V., Brunet, D., Ouvrard, G. & Andre, G. Crystallographic, magnetic and electronic structures of a new layered ferromagnetic compound $Cr_2Ge_2Te_6$. *J. Phys.: Condens. Matter* **7**, 69 (1995).
3. M. Zehetmayer and H. W. Weber, Experimental evidence for a two-band superconducting state of $NbSe_2$ single crystals, *Phys. Rev. B* **82**, 014524 (2010).
4. Xi, X. *et al*. Ising pairing in superconducting $NbSe_2$ atomic layers. *Nat. Phys.* **12**, 139-143 (2016).
5. Bawden, L. *et al*. Spin–valley locking in the normal state of a transition-metal dichalcogenide superconductor. *Nat. Commun.* **7**, 11711 (2016).
